# Supplementary figures and images for: Independent domains for recruitment of PRC1 and PRC2 by human XIST
Source: PLoS Genet. 2021 Mar 22;17(3):e1009123. doi: 10.1371/journal.pgen.1009123 (PMC8016261; doi:10.1371/journal.pgen.1009123)

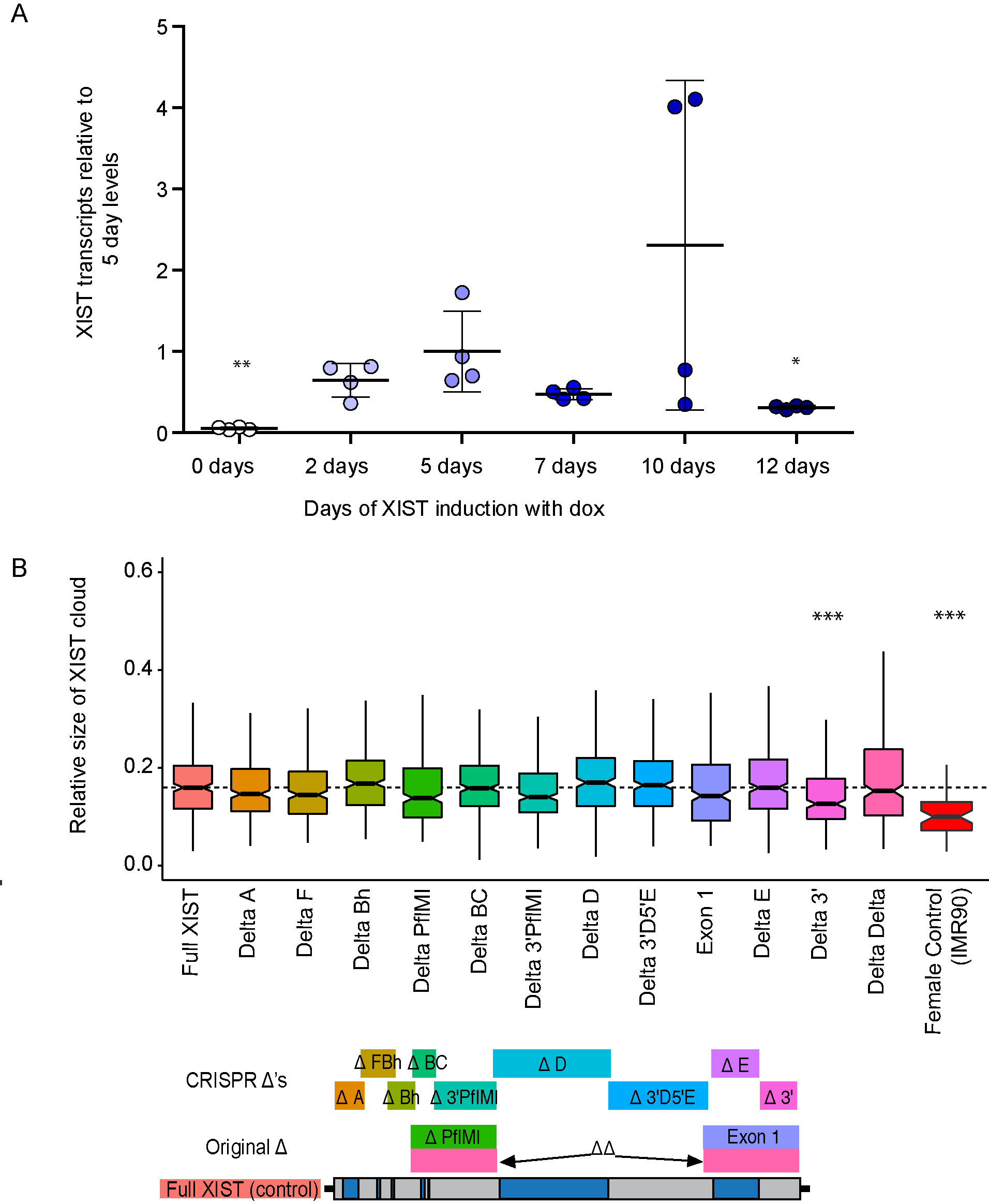

Supplement: S1 Fig — A) XIST transcript levels were measured using RT-qPCR and determining the relative transcript levels compared to the endogenous control gene PGK1. Four biological replicates for each time point of XIST induction in the 8p HT1080 cell line were tested and the relative levels of XIST were normalized to the average level of the 5 day time point, as it was the time point which had become standard in previous examinations of the model system. The mean and standard deviation for each condition are indicated by lines, and individual dots representing the relative expression of each replicate. Increasing darkness of shading was used to indicate increasing length of XIST induction. The statistical significance of a difference between the 5 day treatment and other time points was calculated by two-tailed unpaired t-test (* p < 0.05, ** p< 0.01). B) Approximation of the relative size of XIST RNA clouds across all of the IF-FISH labelled cells analyzed in this paper. For each cell the number of XIST +ve pixels were divided by the absolute number of pixels that could be measured across the length of the cell without intersecting the nucleolar compartments. This information was used to check for any signs of variability in the proportion of the nucleus taken up by XIST RNA. A female control cell line (IMR90) was included in this analysis as a reference however its nuclei was typically much larger than that of the HT1080s, resulting in it occupying a proportionally smaller region of the nuclei. Significance was calculated using M.W. test. (*** p < 7.14x10^-5^). (TIF) [file pgen.1009123.s001.tif]

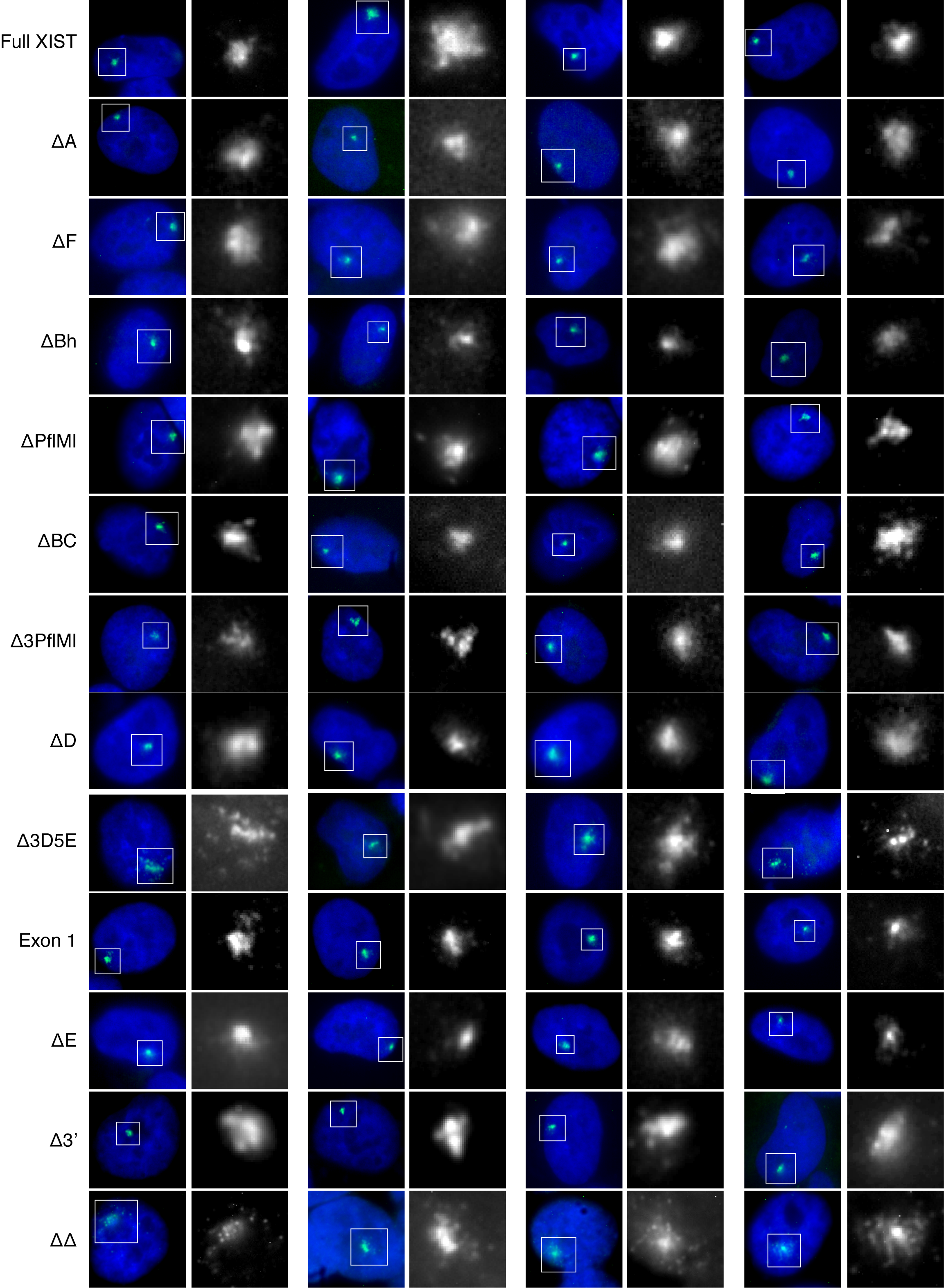

Supplement: S2 Fig — XIST RNA was labelled by FISH in green and the DNA of the nuclei was labelled with DAPI. The white box denotes the area shown in the grayscale image of XIST RNA accompanying each image of a cell nuclei. The greyscale images for each cell show only the fluorescent channel of XIST RNA. (TIF) [file pgen.1009123.s002.tif]

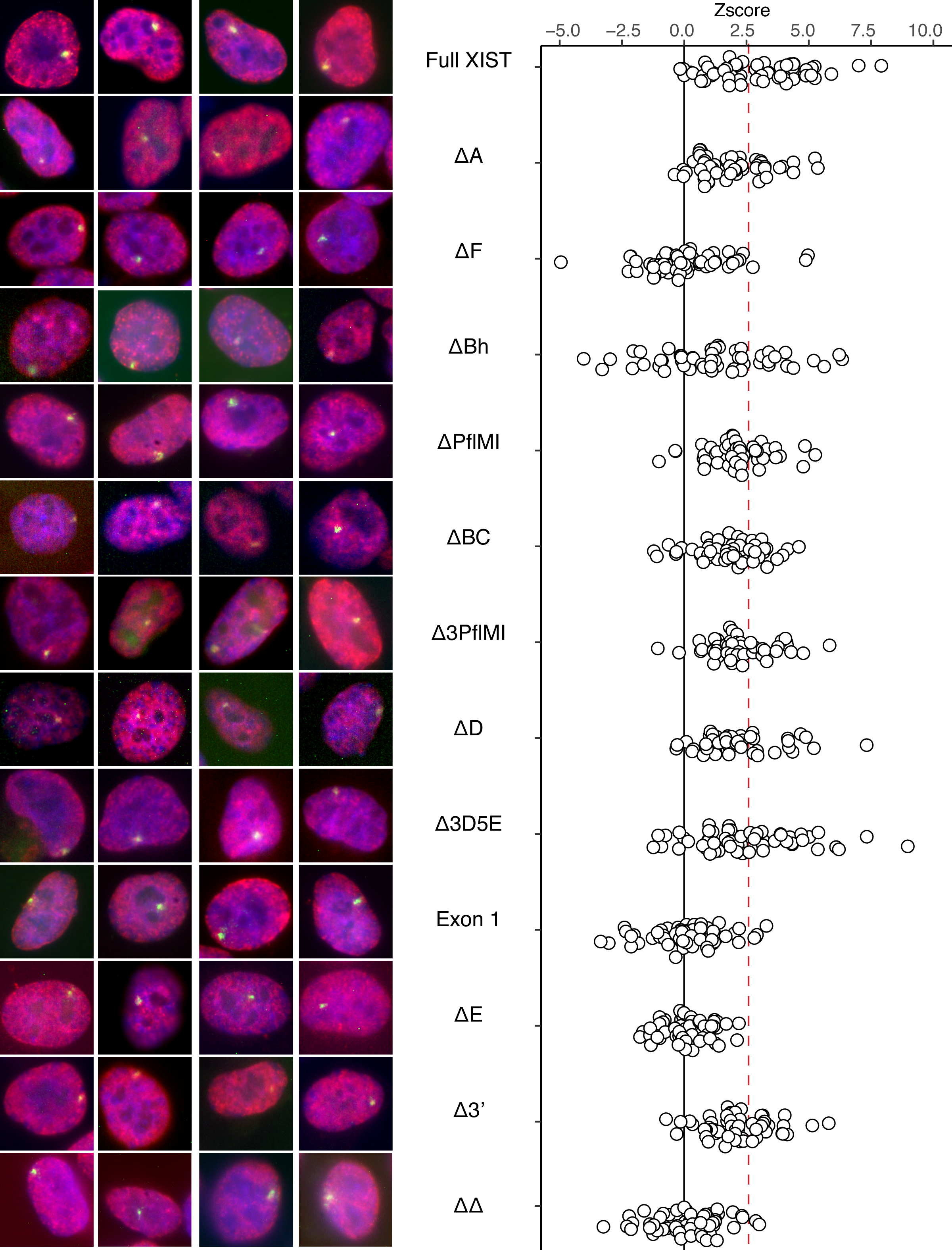

Supplement: S3 Fig — Four example IF-FISH images for each type of XIST inducible construct are in rows to the left of the appropriate label. XIST RNA is labelled green, H3K27me3 labelled red and the DNA labelled blue with DAPI. The z scores calculated for all the analyzed cells for each type of construct are shown in the dot plot, with each cell represented by a single dot. The red dashed line indicates the median z-score (2.59) of the Full XIST construct to provide a point of comparison. (TIF) [file pgen.1009123.s003.tif]

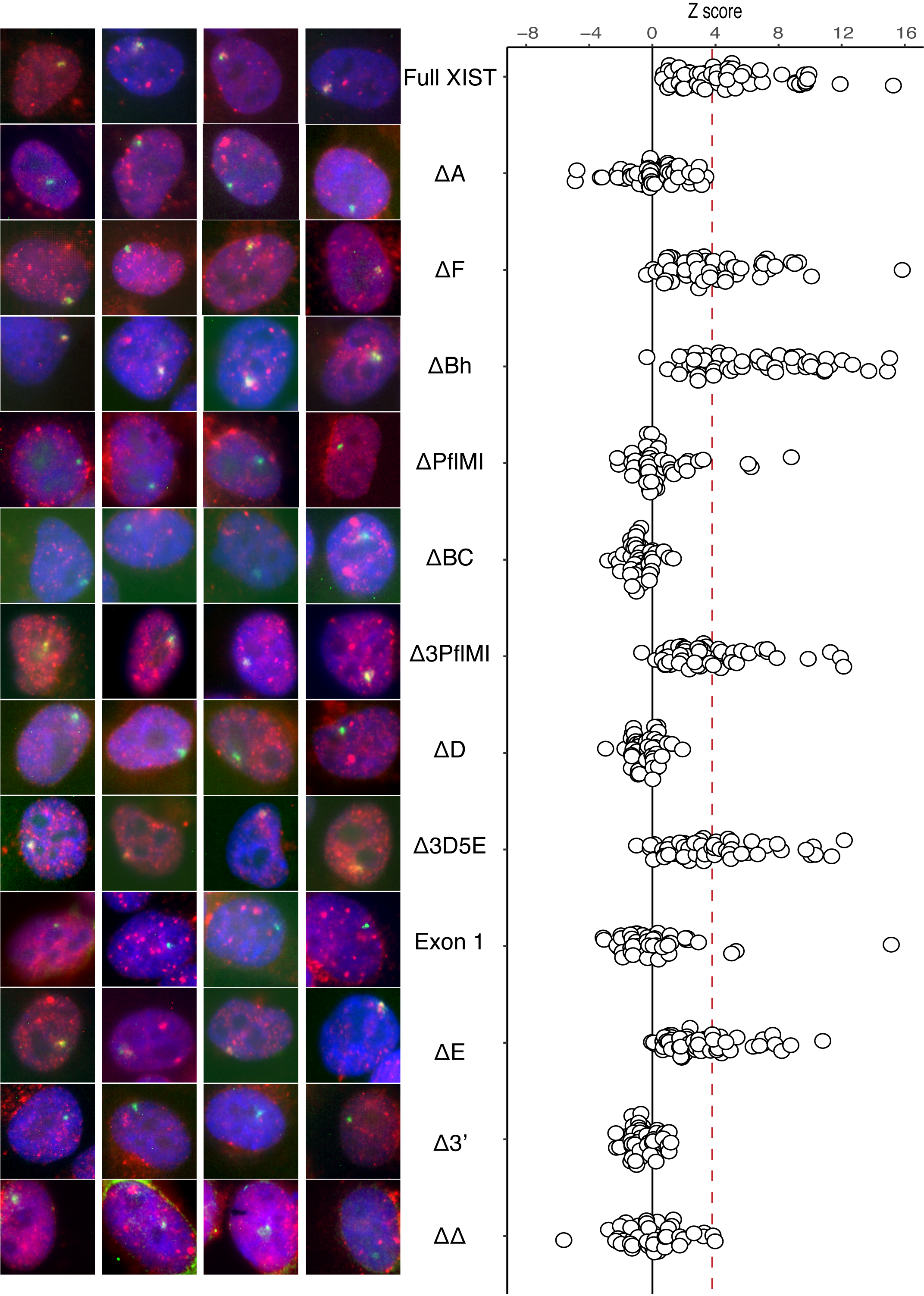

Supplement: S4 Fig — Four example IF-FISH images for each type of XIST inducible construct are in rows to the left of the appropriate label. XIST RNA is labelled green, ubH2A labelled red and the DNA labelled blue with DAPI. The z scores calculated for all the analyzed cells for each type of construct are shown in the dot plot, with each cell represented by a single dot. The red dashed line indicates the median z-score (4.2) of the Full XIST construct to provide a point of comparison. (TIF) [file pgen.1009123.s004.tif]

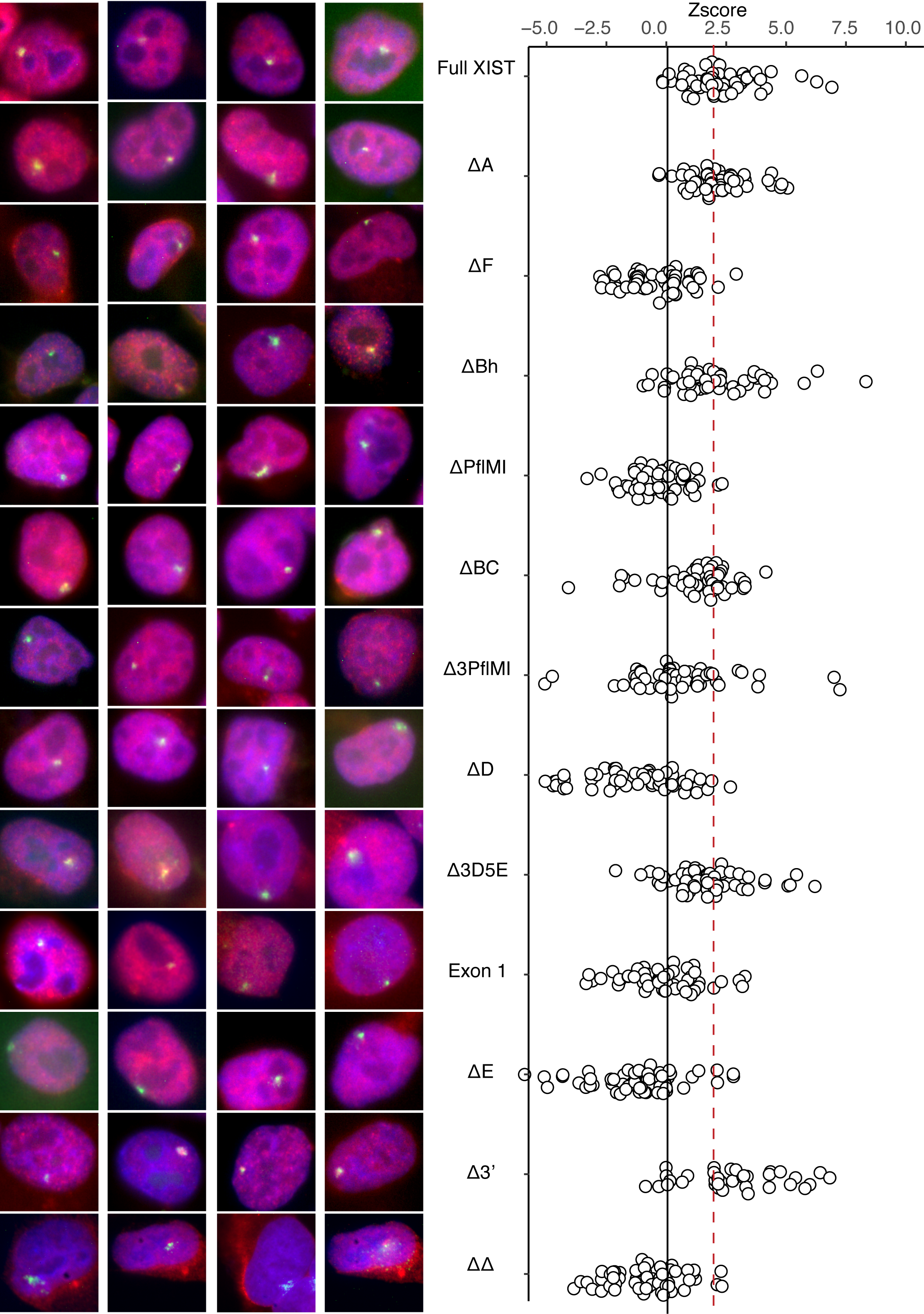

Supplement: S5 Fig — Four example IF-FISH images for each type of XIST inducible construct are in rows to the left of the appropriate label. XIST RNA is labelled green, MacroH2A labelled red and the DNA labelled blue with DAPI. The z scores calculated for all the analyzed cells for each type of construct are shown in the dot plot, with each cell represented by a single dot. The red dashed line indicates the median z-score (2.0) of the Full XIST construct to provide a point of comparison. (TIF) [file pgen.1009123.s005.tif]

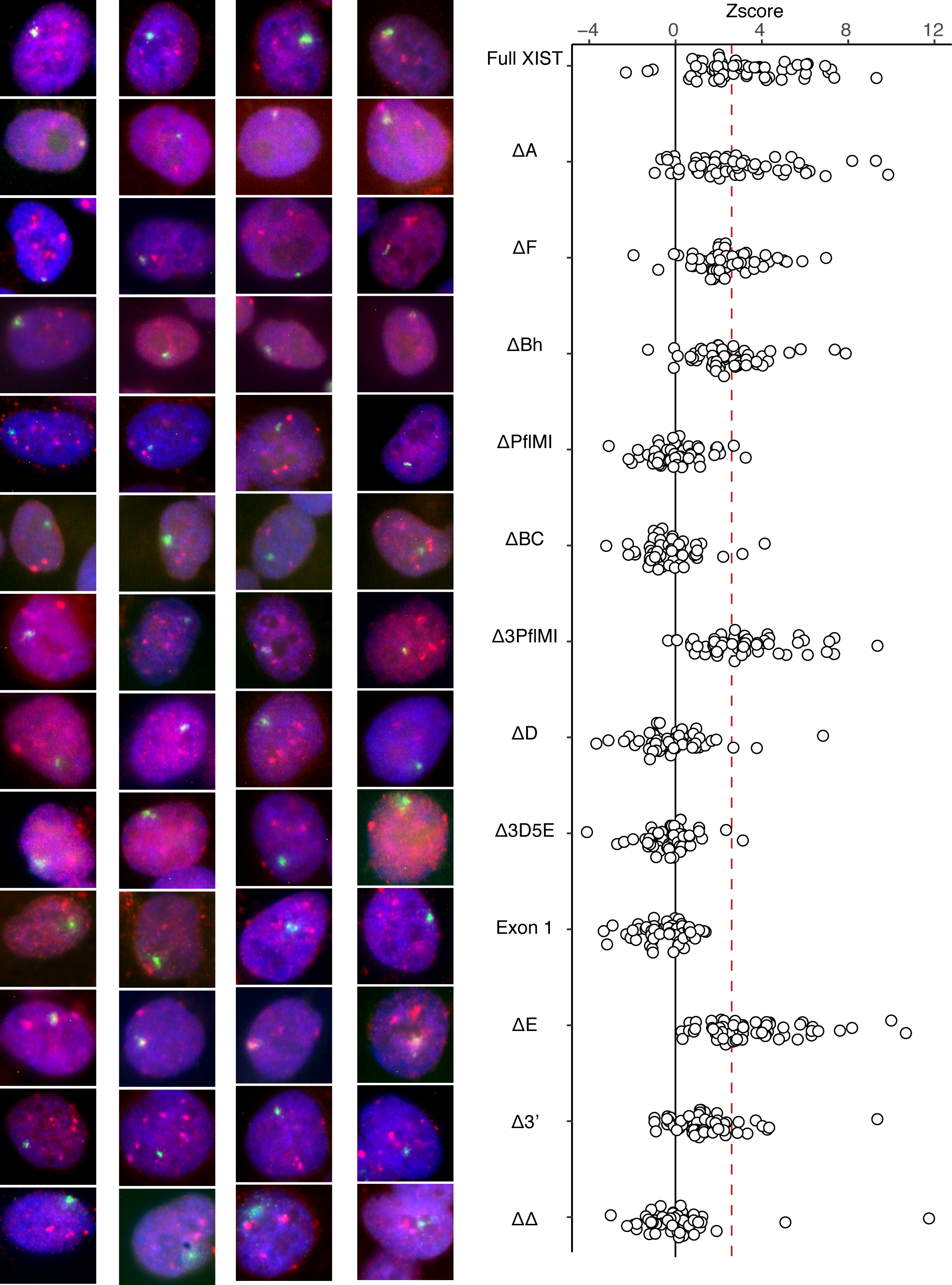

Supplement: S6 Fig — Four example IF-FISH images for each type of XIST inducible construct are in rows to the left of the appropriate label. XIST RNA is labelled green, SMCHD1 labelled red and the DNA labelled blue with DAPI. The z scores calculated for all the analyzed cells for each type of construct are shown in the dot plot, with each cell represented by a single dot. The red dashed line indicates the median z-score (2.7) of the Full XIST construct to provide a point of comparison. (TIF) [file pgen.1009123.s006.tif]

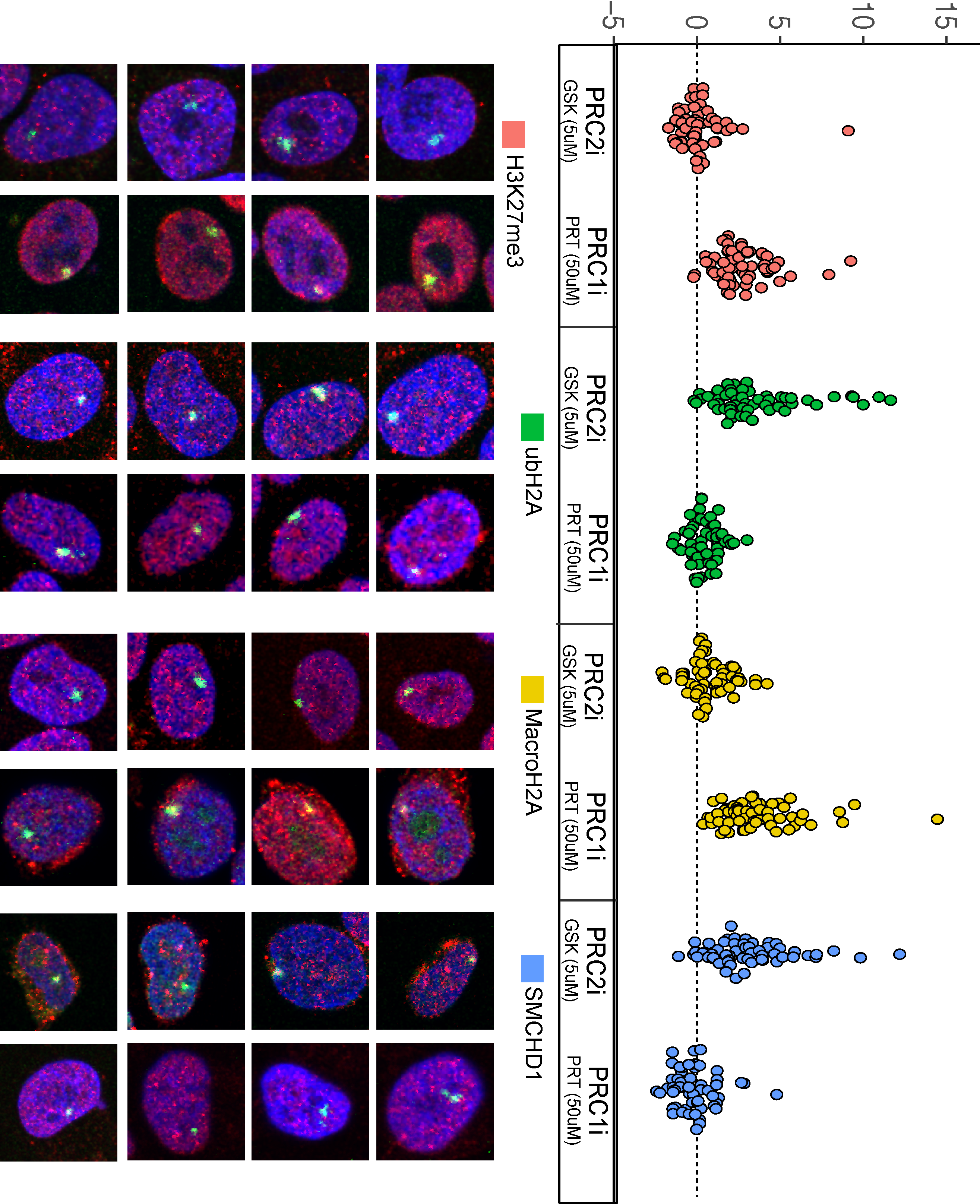

Supplement: S7 Fig — Four example IF-FISH images for each type of XIST inducible construct are in rows to the left of the appropriate label. XIST RNA is labelled green, the heterochromatin features being examined are labelled red and the DNA labelled blue with DAPI. The identity of the heterochromatin marks is adjacent to the photos. The z scores calculated for all the analyzed cells for each type of construct are shown in the dot plot, with each cell represented by a single dot. (TIF) [file pgen.1009123.s007.tif]

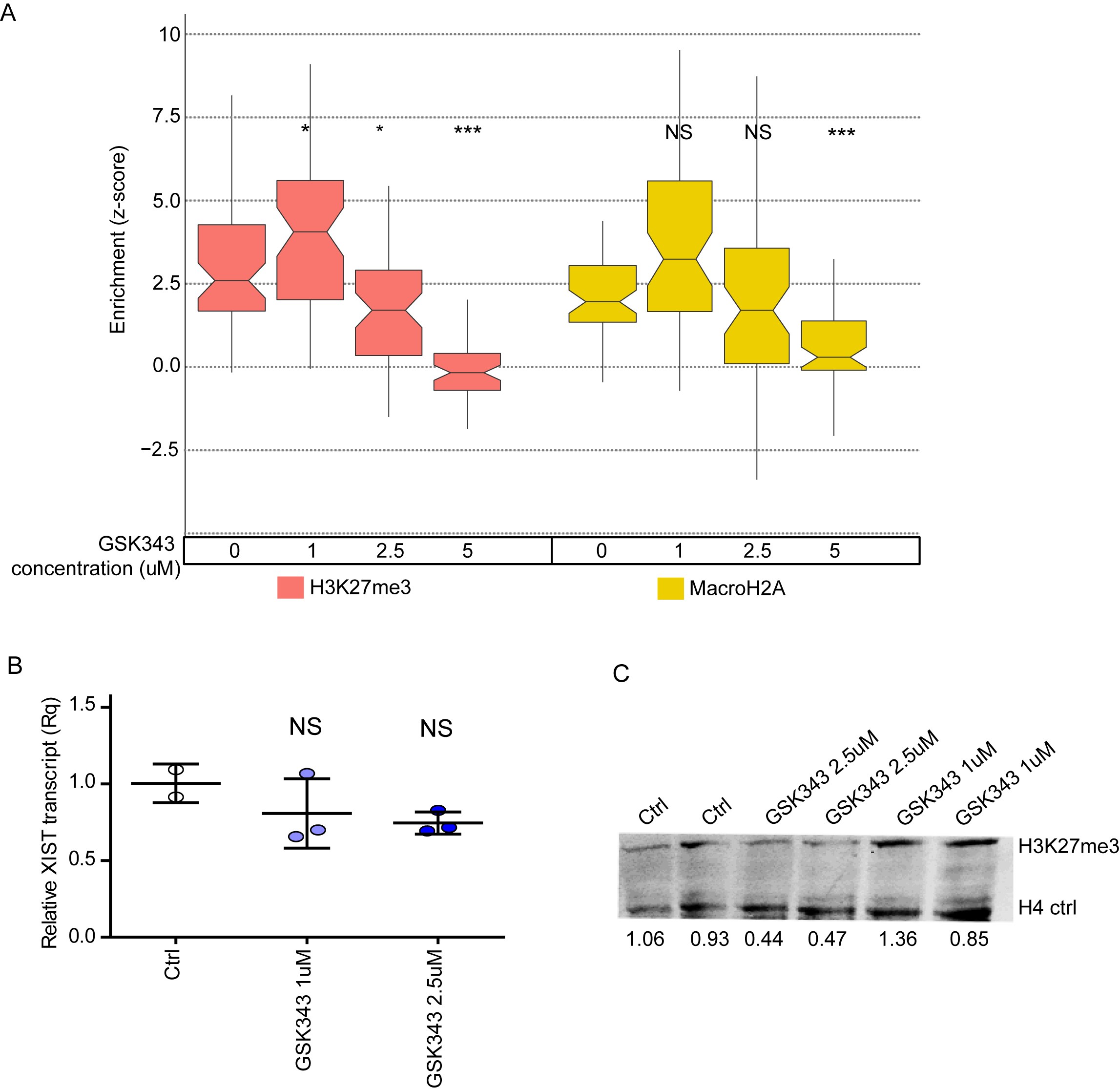

Supplement: S8 Fig — A) The effect of additional PRC2 inhibition with GSK343 oH3K27me3 (pink) and MacroH2A (yellow) at the XIST RNA cloud relative to the average level in the nucleus across a population of 59–61 cells. We were blinded to all cells and chromatin marks identity until after all data and calculations were completed. Statistical significance of the effect of each inhibitor on a chromatin mark was calculated using the Mann Whitney test with adjusted p value (* p < 8.3x10^-3^, ** p < 1.6x10^-3^, *** p < 1.6x10^-4^). B) Relative XIST RNA levels for each inhibitor concentration relative to control 5ddox. The dots denote independent biological replicates and statistical significance was calculated using a t-test (* p < 0.05). C) Western blotting images demonstrating the levels of H3K27me3 after cells had undergone chemical inhibition with GSK343, relative levels of H3K27me3 were determined through comparison with H4 and the normalized values are shown beneath each lane. (TIF) [file pgen.1009123.s008.tif]
