# Supplementary material for: Independent domains for recruitment of PRC1 and PRC2 by human XIST
Source: PLoS Genet. 2021 Mar 22;17(3):e1009123. doi: 10.1371/journal.pgen.1009123 (PMC8016261; doi:10.1371/journal.pgen.1009123)
Supplement: S1 Table — (DOCX) [file pgen.1009123.s009.docx]

### S1 Table: gRNAs used to generate XIST deletions

| **gRNA name** | **Nucleotides into cDNA and strand** | **S-Score** | **E-Score** | **Target Sequence with PAM** | **length** |
| --- | --- | --- | --- | --- | --- |
| XIST gRNA 0.2 | 122 +ve | 100 | 64.9075 | GGACGTGTCAAGAAGACACT AGG | 20 |
| XIST gRNA 0.9 | 887 +ve | 100 | 48.5215 | GTTTGTGCTAAGTTAAACTA GGG | 20 |
| XIST gRNA 1.0 | 900 +ve | 100 | 65.2456 | GTTAAACTAGGGAGGCAAGA TGG | 20 |
| XIST gRNA 1.7 | 1655 +ve | 92.1739 | 42.595 | GCAGCTGTCTTTAGCCAGTC AGG | 20 |
| XIST gRNA 1.9 | 1888 -ve | 100 | 47.4114 | GGGGAGGTATACTTAGCCTT AGG | 20 |
| XIST gRNA 2.1 | 2087 +ve | 94.3478 | 56.2607 | GATGATCGTTGGCCAACAGG TGG | 20 |
| XIST gRNA 2.6 | 2537 +ve | 92.1739 | 54.9581 | GAGTGTTTGAAGGTTTACAC AGG | 20 |
| XIST gRNA 3.1 | 3036 +ve | 100 | 55.1371 | GGACAAAGAATTTCCTTACT CGG | 20 |
| XIST gRNA 3.3 | 3311 -ve | 94.5454 | 42.8973 | GAGTGCTGTCTAATCCAAT GGG | 20 |
| XIST gRNA 5.5 | 5459 +ve | 100 | 67.3981 | GCAGTAATGCAAATGGAGCA AGG | 20 |
| XIST gRNA 6.0 | 5947 -ve | 100 | 54 | GGCCAAGAAATGGGGCCTT AGG | 19 |
| XIST gRNA 8.5 | 8523 -ve | 84.3478 | 58.575 | GCCAAGAAAAGGGGACTTAG GGG | 20 |
| XIST gRNA 8.6 | 8586 -ve | 100 | 54.3169 | GAGGTGGGGCATCCTTGTCT AGG | 20 |
| XIST gRNA 11.9 | 11865 +ve | 100 | 53.7637 | GCCTGGCACTCTAGCACTTG AGG | 20 |
| XIST gRNA 12.2 | 12107 -ve | 100 | 56.0168 | GTGAAAGAAGAGCCACATCT AGG | 20 |
| XIST gRNA 13.7 | 13626 +ve | 100 | 53.4027 | GTTGGGGAAAAAAAAGTGCC AGG | 20 |
| XIST gRNA 13.8 | 13804 +ve | 100 | 66.3505 | GACCACTGCTGGGCAGCAGG AGG | 20 |
| XIST gRNA 14.2 | 14257 +ve | 84.3478 | 65.3497 | GTCACAATTGAAACAAACTG GGG | 20 |
