# Supplementary material for: Independent domains for recruitment of PRC1 and PRC2 by human XIST
Source: PLoS Genet. 2021 Mar 22;17(3):e1009123. doi: 10.1371/journal.pgen.1009123 (PMC8016261; doi:10.1371/journal.pgen.1009123)
Supplement: S2 Table — Each of the cell lines successfully generated for each type of deletion construct are listed along with the gRNAs used to create the deletion and the total number of nucleotides lost from the XIST cDNA sequence. (DOCX) [file pgen.1009123.s010.docx]

### S2 Table: XIST deletion sizes confirmed by sequencing across deletion.

Each of the cell lines successfully generated for each type of deletion construct are listed along with the gRNAs used to create the deletion and the total number of nucleotides lost from the XIST cDNA sequence.

| **Deletion cell line** | **5’ gRNA** | **3’ gRNA** | **Nucleotides deleted** |
| --- | --- | --- | --- |
| Δ A #12 | *XIST* gRNA 0.2 | *XIST* gRNA 0.9 | 777 |
| Δ FBh #21 | *XIST* gRNA 0.9 | *XIST* gRNA 1.9 | 1127 |
| Δ FBh #22 | *XIST* gRNA 1.0 | *XIST* gRNA 1.9 | 811 |
| Δ Bh #5 | *XIST* gRNA 1.7 | *XIST* gRNA 2.6 | 833 |
| Δ Bh #7 | *XIST* gRNA 1.7 | *XIST* gRNA 2.6 | 833 |
| Δ Bh #11 | *XIST* gRNA 1.7 | *XIST* gRNA 2.6 | 857 |
| Δ BC #2 | *XIST* gRNA 2.1 | *XIST* gRNA 3.3 | 1195 |
| Δ BC #8 | *XIST* gRNA 2.1 | *XIST* gRNA 3.3 | 1189 |
| Δ BC #17 | *XIST* gRNA 2.1 | *XIST* gRNA 3.3 | 1195 |
| Δ 3’PflMI #3 | *XIST* gRNA 3.1 | *XIST* gRNA 6.0 | 2859 |
| Δ 3’PflMI #6 | *XIST* gRNA 3.1 | *XIST* gRNA 6.0 | 2859 |
| Δ D #3 | *XIST* gRNA 5.5 | *XIST* gRNA 8.5 | 3084 |
| Δ D #10 | *XIST* gRNA 5.5 | *XIST* gRNA 8.5 | 3092 |
| Δ 3D5E #13 | *XIST* gRNA 8.5 | *XIST* gRNA 12.2 | 3584 |
| Δ 3D5E #14 | *XIST* gRNA 8.5 | *XIST* gRNA 12.2 | 3583 |
| Δ 3D5E #15 | *XIST* gRNA 8.5 | *XIST* gRNA 12.2 | 3588 |
| Δ E #6 | *XIST* gRNA 11.9 | *XIST* gRNA 13.7 | 1844 |
| Δ E #10 | *XIST* gRNA 11.9 | *XIST* gRNA 13.7 | 1778 |
| Δ 3’ #1 | *XIST* gRNA 13.7 | *XIST* gRNA 14.2 | 630 |
| Δ 3’ #7 | *XIST* gRNA 13.7 | *XIST* gRNA 14.2 | 630 |
