# Supplementary material for: Independent domains for recruitment of PRC1 and PRC2 by human XIST
Source: PLoS Genet. 2021 Mar 22;17(3):e1009123. doi: 10.1371/journal.pgen.1009123 (PMC8016261; doi:10.1371/journal.pgen.1009123)
Supplement: S3 Table — The relative size of the XIST +ve region of the nucleus of each cell was measured relative to the maximal length of measurement taken within each cell. The number of cells for each construct along with the mean size of the XIST region and total length measured. The mean relative size of each type of XIST construct within the nucleus and standard deviation (SD) were calculated. Cells from an IMR90 female control cell line were also included. (DOCX) [file pgen.1009123.s011.docx]

### S3 Table: Relative size of XIST RNA cloud in deletion constructs

The relative size of the XIST +ve region of the nucleus of each cell was measured relative to the maximal length of measurement taken within each cell. The number of cells for each construct along with the mean size of the XIST region and total length measured. The mean relative size of each type of XIST construct within the nucleus and standard deviation (SD) were calculated. Cells from an IMR90 female control cell line were also included.

| **Construct** | **# of cells** | **avg. XIST cloud length (pixels)** | **avg. total length (pixels)** | **mean relative size of XIST** | **SD of XIST** |
| --- | --- | --- | --- | --- | --- |
| Full XIST | 301 | 16.189 | 101.578 | 0.1676 | 0.0687 |
| Delta A | 298 | 16.171 | 104.107 | 0.1655 | 0.0810 |
| Delta FBh | 299 | 18.532 | 125.224 | 0.1571 | 0.0705 |
| Delta Bh | 299 | 19.465 | 112.816 | 0.1787 | 0.0779 |
| Delta PflMI | 295 | 15.220 | 101.732 | 0.1597 | 0.0825 |
| Delta BC | 300 | 18.137 | 114.027 | 0.1686 | 0.0730 |
| Delta 3'PflMI | 303 | 18.970 | 128.855 | 0.1556 | 0.0662 |
| Delta D | 288 | 19.319 | 113.010 | 0.1816 | 0.0861 |
| Delta 3'D5'E | 301 | 16.890 | 100.113 | 0.1753 | 0.0771 |
| Exon 1 | 298 | 14.758 | 94.879 | 0.1647 | 0.1008 |
| Delta E | 301 | 18.176 | 109.887 | 0.1731 | 0.0817 |
| Delta 3' | 256 | 15.121 | 112.484 | 0.1450 | 0.0777 |
| Delta Delta | 298 | 15.399 | 87.577 | 0.1832 | 0.1124 |
| Female (IMR90) | 52 | 16.231 | 165.538 | 0.1086 | 0.0552 |
