# Supplementary material for: Independent domains for recruitment of PRC1 and PRC2 by human XIST
Source: PLoS Genet. 2021 Mar 22;17(3):e1009123. doi: 10.1371/journal.pgen.1009123 (PMC8016261; doi:10.1371/journal.pgen.1009123)
Supplement: S4 Table — The relative expression (RQ) of each deletion cell line as well as construct is listed along with the standard deviation (SD) across biological replicates (≥3 per cell line) as well as the resulting p value of any construct or cell lines difference from Full XIST. (DOCX) [file pgen.1009123.s012.docx]

### S4 Table: XIST expression levels in deletion clones relative to Full XIST.

The relative expression (RQ) of each deletion cell line as well as construct is listed along with the standard deviation (SD) across biological replicates (≥3 per cell line) as well as the resulting p value of any construct or cell lines difference from Full XIST.

| **Construct** | **Clone** | **Mean RQ** | **SD** | **p-value** | **Mean** | **SD** | **p-value** |
| --- | --- | --- | --- | --- | --- | --- | --- |
| Δ A | 12 | 0.508 | 0.300 | 1.9E-02 | 0.508 | 0.300 | 1.90E-02 |
| Δ FBh | 21 | 0.678 | 0.425 | 9.6E-02 | 1.044 | 0.686 | 5.71E-01 |
|  | 22 | 1.410 | 0.764 | 4.2E-01 |  |  |  |
| Δ Bh | 5 | 0.971 | 0.319 | 5.3E-01 | 0.813 | 0.315 | 1.01E-01 |
|  | 7 | 0.527 | 0.232 | 4.8E-02 |  |  |  |
|  | 11 | 0.942 | 0.184 | 4.3E-01 |  |  |  |
| Δ PflMI | 3 | 0.630 | 0.171 | 9.0E-02 | 0.630 | 0.171 | 8.96E-02 |
| Δ BC | 2 | 0.653 | 0.235 | 3.1E-02 | 0.773 | 0.272 | 1.71E-02 |
|  | 8 | 0.905 | 0.328 | 3.7E-01 |  |  |  |
|  | 17 | 0.762 | 0.166 | 1.2E-01 |  |  |  |
| Δ 3'PflMI | 3 | 1.147 | 0.460 | 9.1E-01 | 1.150 | 0.489 | 8.88E-01 |
|  | 6 | 1.154 | 0.526 | 9.1E-01 |  |  |  |
| Δ D | 2 | 1.258 | 0.919 | 6.8E-01 | 1.061 | 0.759 | 9.02E-01 |
|  | 10 | 0.864 | 0.360 | 3.0E-01 |  |  |  |
| Δ 3D5E | 13 | 1.249 | 0.919 | 7.0E-01 | 1.075 | 0.857 | 8.31E-01 |
|  | 14 | 1.367 | 1.059 | 5.6E-01 |  |  |  |
|  | 15 | 0.610 | 0.128 | 3.3E-02 |  |  |  |
| Exon 1 | 3 | 1.072 | 0.043 | 8.5E-01 | 1.321 | 0.655 | 4.66E-01 |
|  | 7 | 1.571 | 0.855 | 2.4E-01 |  |  |  |
| Δ E | 6 | 1.762 | 1.085 | 8.1E-02 | 1.797 | 1.017 | 4.02E-02 |
|  | 10 | 1.833 | 0.930 | 3.5E-02 |  |  |  |
| Δ 3' | 1 | 0.365 | 0.115 | 1.1E-03 | 0.395 | 0.169 | 2.04E-05 |
|  | 7 | 0.425 | 0.199 | 1.0E-03 |  |  |  |
| ΔΔ | 12 | 0.986 | 0.739 | 6.4E-01 | 0.986 | 0.739 | 6.40E-01 |
