# Supplementary material for: Independent domains for recruitment of PRC1 and PRC2 by human XIST
Source: PLoS Genet. 2021 Mar 22;17(3):e1009123. doi: 10.1371/journal.pgen.1009123 (PMC8016261; doi:10.1371/journal.pgen.1009123)
Supplement: S7 Table — List of the number of cells analyzed, the median z-score calculated as well as the standard deviation (SD) for each construct. The statistical significance of each population of deletion constructs’ difference from Full XIST in their enrichment was calculated using the Mann-Whitney U test and the p values are listed. (DOCX) [file pgen.1009123.s015.docx]

### S7 Table: Summary of MacroH2A enrichment in deletion constructs.

List of the number of cells analyzed, the median z-score calculated as well as the standard deviation (SD) for each construct. The statistical significance of each population of deletion constructs’ difference from Full XIST in their enrichment was calculated using the Mann-Whitney U test and the p values are listed.

| MacroH2A | number of cells | median z-score | sd | MW p-value |
| --- | --- | --- | --- | --- |
| Full XIST | 60 | 1.958 | 1.469 | -- |
| Δ A | 58 | 2.213 | 2.227 | 6.26E-01 |
| Δ FBh | 59 | -0.434 | 1.189 | 5.34E-17 |
| Δ Bh | 60 | 1.709 | 2.639 | 2.90E-01 |
| Δ PflMI | 60 | -0.348 | 1.124 | 1.89E-17 |
| Δ BC | 60 | 1.562 | 2.247 | 8.62E-03 |
| Δ 3'PflMI | 60 | 0.313 | 2.197 | 3.06E-09 |
| Δ D | 60 | -1.042 | 2.338 | 4.25E-17 |
| Δ 3D5E | 60 | 1.541 | 1.444 | 1.09E-01 |
| Exon 1 | 59 | -0.096 | 1.380 | 9.44E-15 |
| Δ E | 60 | -1.173 | 1.784 | 6.08E-17 |
| Δ 3' | 60 | 2.609 | 2.288 | 7.58E-02 |
| ΔΔ | 61 | -0.737 | 1.731 | 3.76E-18 |
