# Supplementary material for: Independent domains for recruitment of PRC1 and PRC2 by human XIST
Source: PLoS Genet. 2021 Mar 22;17(3):e1009123. doi: 10.1371/journal.pgen.1009123 (PMC8016261; doi:10.1371/journal.pgen.1009123)
Supplement: S10 Table — List of the number of cells analyzed, the median z-score calculated as well as the standard deviation (SD) for each treatment condition and heterochromatin feature. The statistical significance of each population of inhibitor treated cells’ difference from the uninhibited control population was calculated using the Mann-Whitney U test and the p values are listed. (DOCX) [file pgen.1009123.s018.docx]

### S10 Table: Summary of effect of additional doses of GSK343 treatment on XIST mediated chromatin remodelling.

List of the number of cells analyzed, the median z-score calculated as well as the standard deviation (SD) for each treatment condition and heterochromatin feature. The statistical significance of each population of inhibitor treated cells’ difference from the uninhibited control population was calculated using the Mann-Whitney U test and the p values are listed.

| Treatment | Mark | number of cells | median z-score | sd | MW p-value |
| --- | --- | --- | --- | --- | --- |
| Control | H3K27me3 | 60 | 2.593 | 1.704 |  |
| GSK343 1uM | H3K27me3 | 60 | 4.29074069 | 2.963 | 0.002501 |
| GSK343 2.5uM | H3K27me3 | 60 | 1.83969447 | 2.090 | 0.006302 |
| GSK343 5uM | H3K27me3 | 58 | -0.09035036 | 1.479 | 7.24E-16 |
| Control | MacroH2A | 60 | 1.958 | 1.469 |  |
| GSK343 1uM | MacroH2A | 60 | 3.15001376 | 2.486 | 0.011074 |
| GSK343 2.5uM | MacroH2A | 60 | 1.62133624 | 2.536 | 0.203881 |
| GSK343 5uM | MacroH2A | 60 | 0.22405691 | 1.230 | 1.30E-10 |
