# Supplementary material for: Independent domains for recruitment of PRC1 and PRC2 by human XIST
Source: PLoS Genet. 2021 Mar 22;17(3):e1009123. doi: 10.1371/journal.pgen.1009123 (PMC8016261; doi:10.1371/journal.pgen.1009123)
Supplement: S12 Table — (DOCX) [file pgen.1009123.s020.docx]

### S12 Table: List of Antibodies used for Immunofluorescence and western blotting.

| Name | Company | Catalog # | Host species |
| --- | --- | --- | --- |
| Anti-ubiquitin-Histone H2A Antibody | Sigma-Aldrich | 05-678 | mouse |
| Anti-H4 Antibody | Upstate | 25296 | rabbit |
| β-actin loading control antibody | Invitrogen | MA5-15739 | mouse |
| H3K27me3 Antibody | Diagenode | C15410069 | rabbit |
| Anti-Histone Macro H2A Antibody | Upstate | 07-219 | rabbit |
| Anti-SMCHD1 antibody | Abcam | ab31865 | rabbit |
| Goat anti-Rabbit IgG (H+L) Cross-Adsorbed Secondary Antibody, Alexa Fluor 594 | Invitrogen | A11012 | goat |
| Goat anti-Mouse IgG (H+L) Cross-Adsorbed Secondary Antibody, Alexa Fluor 594 | Invitrogen | A11005 | goat |
| IRDye 680RD Goat anti-Rabbit IgG | Licor | 925-68071 | goat |
| IRDye 800CW Goat anti-Mouse IgG | Licor | 925-32210 | goat |
