# Supplementary material for: Independent domains for recruitment of PRC1 and PRC2 by human XIST
Source: PLoS Genet. 2021 Mar 22;17(3):e1009123. doi: 10.1371/journal.pgen.1009123 (PMC8016261; doi:10.1371/journal.pgen.1009123)
Supplement: S1 Information — The sequencing results across the cut size of each of the deletion constructs created through CRISPR were listed below arranged from 5’ to 3’ along the sense sequence of the XIST construct. Bolded letters indicated the gRNA target sequences remaining within the final transcript. The cut site was denoted by vertical bars (|) which encapsulated the summary of the deletion size and any other characteristics of note. The sequences of the repeats were underlined to provide landmarks when referring to the location of the deletions in each instance. (DOCX) [file pgen.1009123.s021.docx]

List of sequences across the cut sites of CRISPR deletion constructs

The sequencing results across the cut size of each of the deletion constructs created by CRISPR were listed below arranged from 5’ to 3’ along the sense sequence of the *XIST* construct. Bolded letters indicated the gRNA target sequences remaining within the final transcript. The cut site was denoted by vertical bars ( | ) which encapsulated the summary of the deletion size and any other characteristics of note. The sequences of the repeats were underlined to provide landmarks when referring to the location of the deletions in each instance.

Δ A #12 Sequencing Results

(start of *XIST* cDNA) TCTAGAACATTTTCTAGTCCCCCAACACCCTTTATGGCGTATTTCTTTAAAAAAATCACCTAAATTCCATAAAATATTTTTTTAAATTCTATACTTTCTCCT**AGTG**

|777 nucleotides removed from full length sequence|

**ACTA**GGGAGGCAAGATGGATGATAGCAGGTCAGGCAGAGGAAGTCATGTGCATTGCATGAGCTAAACCTAT

Δ FBh #21

TTGGTTTTGTGGGTTGTTGCACTCTCTGGAATATCTACACTTTTTTTTGCTGCTGATCATTTGGTGGTGTGTGAGTGTACCTACCGCTTTGGCAGAGAATGACTCTGCAGTTAAGCTAAGGGCGTGTTCAGATTGTGGAGGAAAAGTGGCCGCCATTTTAGACTTGCCGCATAACTCGGCTTAGGGCTAGTC**GTTTGTGCTAAGT**

|1127 nucleotides removed from full length sequence|

TCCAGGCCTGCTTGGTGTGGACATGGTGGTGAGCCGTGGCAAGGACCAGAATGGATCACAGATGATCGTTGGCCAACAGGTGGCAGAAGAGGAATTCCTGCCTTCCTCAAGAGGAACACCTACCCCTTGGCTAATGCTGGGGTCGGATTTTGATTTATATTTATCTTTTGGATGTCAGTCATACAGTCTGATTTTGTG

Δ FBh #22

AAGAATCATCTTTTATCAGTACAAGGGACTAGTTAAAAATGGAAGGTTAGGAAAGACTAAGGTGCAGGGCTTAAAATGGCGATTTTGACATTGCGGCATTGCTCAGCATGGCGGGCTGTGCTTTGTTAGGTTGTCCAAAATGGCGGATCCAGTTCTGTCGCAGTGTTCAAGTGGCGGGAAGGCC

|811 nucleotides removed from full length sequence|

AGGCCTGCTTGGTGTGGACATGGTGGTGAGCCGTGGCAAGGACCAGAATGGATCACAGATGATCGTTGGCCAACAGGTGGCAGAAGAGGAATTCCTGCCTTCCTCAAGAGGAACACCTACCCCTTGGCTAATGCTGGGGTCGGATTTTGATTTATATTTATCTTTTGGATGTCAGTCATACAGTCTGATTTTGTG

Δ Bh #5

GCTTGCCGCATTGTTAAAGATGGCGGGTTTTGCCGCCTAGTGCCACGCAGAGCGGGAGAAAAGGTGGGATGGACAGTGCTGGATTGCTGCATAACCCAACCAATTAGAAATGGGGGTGGAATTGATCACAGCCAATTAGAGCAGAAGATGGAATTAGACTGATGACACACTGTCCAGCTACTCAGCGAAGACCTGGGTGAATTAGCATGGCACTTC**GCAGCTGTCTTTAGCCA**

|833 nucleotides removed from full length sequence|

**CAC**AGGTCCAGGCCTTGCTTTGTTCCCATCCTTGATGCTGCACTAATTGACTAATCACCTACTTATCAGACAGGAAACTTGAATTGCTGTGGTCTGGTGTCCTCTATTCAGACTTATTATATTGGAGTATTTCAATTTTTCGTTGTATCCTGCCTGCCTAGCATCCAGTTCCTCCCCAGCCCTGCTCCCAGCAAACCCCTAGTCTAGCCCCAGCCCTACTCCCACCCCGCCCCAGCCCTGCCCCAGCC

Δ Bh #7

GCTTGCCGCATTGTTAAAGATGGCGGGTTTTGCCGCCTAGTGCCACGCAGAGCGGGAGAAAAGGTGGGATGGACAGTGCTGGATTGCTGCATAACCCAACCAATTAGAAATGGGGGTGGAATTGATCACAGCCAATTAGAGCAGAAGATGGAATTAGACTGATGACACACTGTCCAGCTACTCAGCGAAGACCTGGGTGAATTAGCATGGCACTTC**GCAGCTGTCTTTAGCCA**

|833 nucleotides removed from full length sequence|

**CAC**AGGTCCAGGCCTTGCTTTGTTCCCATCCTTGATGCTGCACTAATTGACTAATCACCTACTTATCAGACAGGAAACTTGAATTGCTGTGGTCTGGTGTCCTCTATTCAGACTTATTATATTGGAGTATTTCAATTTTTCGTTGTATCCTGCCTGCCTAGCATCCAGTTCCTCCCCAGCCCTGCTCCCAGCAAACCCCTAGTCTAGCCCCAGCCCTACTCCCACCCCGCCCCAGCCCTGCCCCAGCC

Δ Bh #11

GCTTGCCGCATTGTTAAAGATGGCGGGTTTTGCCGCCTAGTGCCACGCAGAGCGGGAGAAAAGGTGGGATGGACAGTGCTGGATTGCTGCATAACCCAACCAATTAGAAATGGGGGTGGAATTGATCACAGCCAATTAGAGCAGAAGATGGAATTAGACTGATGACACACTGTCCAGCTACTCAGCGAAGACCTGGGTGAATTAGCATGGCACTTC**GCAG**

|857 nucleotides removed from full length sequence|

GCCTTGCTTTGTTCCCATCCTTGATGCTGCACTAATTGACTAATCACCTACTTATCAGACAGGAAACTTGAATTGCTGTGGTCTGGTGTCCTCTATTCAGACTTATTATATTGGAGTATTTCAATTTTTCGTTGTATCCTGCCTGCCTAGCATCCAGTTCCTCCCCAGCCCTGCTCCCAGCAAACCCCTAGTCTAGCCCCAGCCCTACTCCCACCCCGCCCCAGCCCTGCCCCAGCC

Δ BC #2

TTGGTGCCTCACCTAAGGCTAAGTATACCTCCCCCCCCACCCCCCAACCCCCCCAACTCCCCACCCCCACCCCCCACCCCCCACCTCCCCACCCCCCTACCCCCCTACCCCCCTACCCCCCTCTGGTCTGCCCTGCACTGCACTGTTGCCATGGGCAGTGCTCCAGGCCTGCTTGGTGTGGACATGGTGGTGAGCCGTGGCAAGGACCAGAATGGATCACA**GATGATCGTTG**

|1195 nucleotides removed from full length sequence plus novel 141 nucleotide novel sequence integrated during repair|

**TTGGATTAGACAGCACTC**TGAACCCCATTTGCATTCAGCAGGGGGTCGCAGACAACCCGTCTTTTGTTGGACAGTTAAAATGCTCAGTCCCAATTGTCATAGCTTTGCCTATTAAACAAAGGCACCCTACTGCGCTTTTTGCTGTGCTTCTGGAGAATCCTGCTGTTCTTGGACAATTAAAGAACAAAGTAGTAATTGCTAATTGTCTCACCCATTAATCATGAAGACTA

Δ BC #8

TTGGTGCCTCACCTAAGGCTAAGTATACCTCCCCCCCCACCCCCCAACCCCCCCAACTCCCCACCCCCACCCCCCACCCCCCACCTCCCCACCCCCCTACCCCCCTACCCCCCTACCCCCCTCTGGTCTGCCCTGCACTGCACTGTTGCCATGGGCAGTGCTCCAGGCCTGCTTGGTGTGGACATGGTGGTGAGCCGTGGCAAGGACCAGAATGGATCACA**GATGATCGTTGGCCA**

|1189 nucleotides removed from full length sequence|

**GGATTAGACAGCACTC**TGAACCCCATTTGCATTCAGCAGGGGGTCGCAGACAACCCGTCTTTTGTTGGACAGTTAAAATGCTCAGTCCCAATTGTCATAGCTTTGCCTATTAAACAAAGGCACCCTACTGCGCTTTTTGCTGTGCTTCTGGAGAATCCTGCTGTTCTTGGACAATTAAAGAACAAAGTAGTAATTGCTAATTGTCTCACCCATTAATCATGAAGACTA

Δ BC #17

TTGGTGCCTCACCTAAGGCTAAGTATACCTCCCCCCCCACCCCCCAACCCCCCCAACTCCCCACCCCCACCCCCCACCCCCCACCTCCCCACCCCCCTACCCCCCTACCCCCCTACCCCCCTCTGGTCTGCCCTGCACTGCACTGTTGCCATGGGCAGTGCTCCAGGCCTGCTTGGTGTGGACATGGTGGTGAGCCGTGGCAAGGACCAGAATGGATCACA**GATGATCGTTG**

|1195 nucleotides removed from full length sequence plus novel 145nucleotide novel sequence integrated during repair|

**TTGGATTAGACAGCACTC**TGAACCCCATTTGCATTCAGCAGGGGGTCGCAGACAACCCGTCTTTTGTTGGACAGTTAAAATGCTCAGTCCCAATTGTCATAGCTTTGCCTATTAAACAAAGGCACCCTACTGCGCTTTTTGCTGTGCTTCTGGAGAATCCTGCTGTTCTTGGACAATTAAAGAACAAAGTAGTAATTGCTAATTGTCTCACCCATTAATCATGAAGACTA

Δ 3’PflMI #3

CCCAGTCCCCTAACCCCCCAGCCCTAGCCCCAGTCCCAGTCCTAGTTCCTCAGTCCCGCCCAGCTTCTCTCGAAAGTCACTCTAATTTTCATTGATTCAGTGCTCAAAATAAGTTGTCCATTGCTTATCCTATTATACTGGGATATTCCGTTTACCCTTGGCATTGCTGATCTTCAGTACTGACTCCTTGACCATTTTCAGTTAATGCATACAATCCCATTTGTCTGTGATCTCA**GGACAAAGAATTTCCTT**

|2859 nucleotides removed from full length sequence|

**GCCCCATTTCTTGGCC**TCCCAATATGTGTGATTGTATTTGTCGAGGTTGCTATGCACTAGAGAAGGAAAGTGCTCCCCTCATCCCCACTTTTCCCTTCCAGCAGGAAGTGCCCACCCCATAAGACCCTTTTATTTGGAGAGTCTAGGTGCACAATTGTAAGTGACCACAAGCATGCATCTTGGACATTTATGTGCGTAATCGCACACTGCTCATTCCATGTGAATAAGGTCCTACTCTCCGACCCCTT

Δ 3’PflMI #6

CCCAGTCCCCTAACCCCCCAGCCCTAGCCCCAGTCCCAGTCCTAGTTCCTCAGTCCCGCCCAGCTTCTCTCGAAAGTCACTCTAATTTTCATTGATTCAGTGCTCAAAATAAGTTGTCCATTGCTTATCCTATTATACTGGGATATTCCGTTTACCCTTGGCATTGCTGATCTTCAGTACTGACTCCTTGACCATTTTCAGTTAATGCATACAATCCCATTTGTCTGTGATCTCA**GGACAAAGAATTTCCTT**

|2859 nucleotides removed from full length sequence|

**GCCCCATTTCTTGGCC**TCCCAATATGTGTGATTGTATTTGTCGAGGTTGCTATGCACTAGAGAAGGAAAGTGCTCCCCTCATCCCCACTTTTCCCTTCCAGCAGGAAGTGCCCACCCCATAAGACCCTTTTATTTGGAGAGTCTAGGTGCACAATTGTAAGTGACCACAAGCATGCATCTTGGACATTTATGTGCGTAATCGCACACTGCTCATTCCATGTGAATAAGGTCCTACTCTCCGACCCCTT

Δ D #3

GCCTGGCCAACATTAAAAAAAAAAAAAAGTAAGACAATTGCCCTGGAATCCCATCCCCCTCACACCTCCTTGGCAAAGCAGCAGGAGTGCTAACTAGCTAGTGCTTCTTCTCTTATACTGCTTAAATGCGCATAATTAGCAGTAGTTGATGTGCCCCTATGTTAGAGTAGAATCCCGCTTCCT

|3084 nucleotides including D repeat removed from full length sequence|

TTGTTGACATGCATAATTGCATTTATGTTGGTTCTTGTGCCCTAGACAAGGATGCCCCACCTCTTTTCAATAGTGGGTGCCCACTCCTTATGATCTTTACATTTGAACAGTTAATGTGAATAATTGCAGTTGTCCACAACCCTATCACTTCTAGGACCATTATACCTCTTTTGCATTACTGTGGGGTATACTGTTTCCCTCCAAGGCCCCTTCTGGTGGACTATCAACATAT

Δ D #10

GCCTGGCCAACATTAAAAAAAAAAAAAAGTAAGACAATTGCCCTGGAATCCCATCCCCCTCACACCTCCTTGGCAAAGCAGCAGGAGTGCTAACTAGCTAGTGCTTCTTCTCTTATACTGCTTAAATGCGCATAATTAGCAGTAGTTGATGTGCCCCTATGTTAGAGTAGAATCC

|3092 nucleotides including D repeat removed from full length sequence|

**CCTTTTCTTGG**CTTGTTGACATGCATAATTGCATTTATGTTGGTTCTTGTGCCCTAGACAAGGATGCCCCACCTCTTTTCAATAGTGGGTGCCCACTCCTTATGATCTTTACATTTGAACAGTTAATGTGAATAATTGCAGTTGTCCACAACCCTATCACTTCTAGGACCATTATACCTCTTTTGCATTACTGTGGGGTATACTGTTTCCCTCCAAGGCCCCTTCTGGTGG

Δ 3D5E #13

TTTTAAGATTCTTATATTTGTCCAAAGTACATGGTTTTAATTGACCACAACAATGTCCCTTGGACATTAATGTATGTAATCACCACATGGTTCATCCTAATTAAACAAAGTTCTACCTTCTCACCCTCCATTTGCAGTATACCAGGGTTGCTGACCCCC**TA**

|3584 nucleotides removed from full length sequence|

**TGTGGCTCTTCTTTCAC**GCTTTATTTCATGTCTCCTTTTTGGGTCACATGCTGTGTGCTTTTTGTCCTTTTCTTGTTCTGTCTACCTCTCCTTTCTCTGCCTACCTCTC

Δ 3D5E #14

TTTTAAGATTCTTATATTTGTCCAAAGTACATGGTTTTAATTGACCACAACAATGTCCCTTGGACATTAATGTATGTAATCACCACATGGTTCATCCTAATTAAACAAAGTTCTACCTTCTCACCCTCCATTTGCAGTATACCAGGGTTGCTGACCCCC**TAAG**

|3583 nucleotides removed from full length sequence|

**GTGGCTCTTCTTTCAC**GCTTTATTTCATGTCTCCTTTTTGGGTCACATGCTGTGTGCTTTTTGTCCTTTTCTTGTTCTGTCTACCTCTCCTTTCTCTGCCTACCTCTC

Δ 3D5E #15

TTTTAAGATTCTTATATTTGTCCAAAGTACATGGTTTTAATTGACCACAACAATGTCCCTTGGACATTAATGTATGTAATCACCACATGGTTCATCCTAATTAAACAAAGTTCTACCTTCTCACCCTCCATTTGCAGTATACCAGGGTTGCTGACCCCC**TAA**

|3588 nucleotides removed from full length sequence|

**CTCTTCTTTCAC**GCTTTATTTCATGTCTCCTTTTTGGGTCACATGCTGTGTGCTTTTTGTCCTTTTCTTGTTCTGTCTACCTCTCCTTTCTCTGCCTACCTCTC

Δ E #6

CTGGGCAACAACCCTAGGTCAGGAGGTTCTGTCAAGATACTTTCCTGGTCCCAGATAGGAAGATAAAGTCTCAAAAACAACCACCACACGTCAAGCTCTTCATTGTTCCTATCTGCCAAATCATTATACTTCCTACAAGCAGTGCAGAGAGCTGAGTCTTCAGCAGGTCCAAGAAATT

|1844 nucleotides removed from full length sequence plus novel GACC sequence integrated into sequence|

TGTGAAGAGATGCTCCAGGCCAATGAGAAGAATTAGACAAGAAATACACAGATGTGCCAGACTTCTGAGAAGCACCTGCCAGCAACAGCTTCCTTCTTTGA GCTTAGGTGAG

Δ E #10

CTGGGCAACAACCCTAGGTCAGGAGGTTCTGTCAAGATACTTTCCTGGTCCCAGATAGGAAGATAAAGTCTCAAAAACAACCACCACACGTCAAGCTCTTCATTGTTCCTATCTGCCAAATCATTATACTTCCTACAAGCAGTGCAGAGAGCTGAGTCTTCAGCAGGTCCAAGAAATTTGAACACACTGAAGGAAGTCAGCCTTCCCACCTGAAGATCAACA

|1778 nucleotides removed from full length sequence|

**TGCC**AGGCTCTCTAGAGAAAAATGTGAAGAGATGCTCCAGGCCAATGAGAAGAATTAGACAAGAAATACACAGATGTGCCAGACTTCTGAGAAGCACCTGCCAGCAACAGCTTCCTTCTTTGA GCTTAGGTGAGC

Δ 3’ #1

TGTTAACTTCCCTCAGGAGCAGACATTCATATAGGTGATACTGTATTTCAGTCCTTTCTTTTGACCCCAGAAGCCCTAGACTGAGAAGATAAAATGGTCAGGTT**GTTGGGGAAAAAAAAGT**

|630 nucleotides removed from full length sequence|

**ACT**GGGGAGTTGGTTGCTATTGTAAAATAAAATATACTGTTTTGAAA (end of *XIST* cDNA)

Δ 3’ #7

TGTTAACTTCCCTCAGGAGCAGACATTCATATAGGTGATACTGTATTTCAGTCCTTTCTTTTGACCCCAGAAGCCCTAGACTGAGAAGATAAAATGGTCAGGTT**GTTGGGGAAAAAAAAGT**

|630 nucleotides removed from full length sequence|

**ACT**GGGGAGTTGGTTGCTATTGTAAAATAAAATATACTGTTTTGAAA (end of *XIST* cDNA)
