# Supplementary material for: Independent domains for recruitment of PRC1 and PRC2 by human XIST
Source: PLoS Genet. 2021 Mar 22;17(3):e1009123. doi: 10.1371/journal.pgen.1009123 (PMC8016261; doi:10.1371/journal.pgen.1009123)
Supplement: S2 Information — The sequences of XIST absent from both constructs aiming to examine the effect of loss of repeat A on the activity of XIST are listed. The names of the construct are shown in bold along with the nucleotide positions within the XIST transcript that have been removed. The sequence corresponding to Repeat A has been underlined. (DOCX) [file pgen.1009123.s022.docx]

# Sequence removed from CRISPR generated Delta A#12 construct and previously generated Delta XB

The sequences of XIST absent from both constructs aiming to examine the effect of loss of repeat A on the activity of XIST are listed. The names of the construct are shown in bold along with the nucleotide positions within the XIST transcript that have been removed. The sequence corresponding to Repeat A has been underlined.

**Delta A #12 Deleted Sequence** (106 to 885bp, Repeat A underlined)

TCTTCTTGACACGTCCTCCATATTTTTTTAAAGAAAGTATTTGGAATATTTTGAGGCAATTTTTAATATTTAAGGAATTTTTCTTTGGAATCATTTTTGGTTGACATCTCTGTTTTTTGTGGATCAGTTTTTTACTCTTCCACTCTCTTTTCTATATTTTGCCCATCGGGGCTGCGGATACCTGGTTTTATTATTTTTTCTTTGCCCAACGGGGCCGTGGATACCTGCCTTTTAATTCTTTTTTATTCGCCCATCGGGGCCGCGGATACCTGCTTTTTATTTTTTTTTCCTTAGCCCATCGGGGTATCGGATACCTGCTGATTCCCTTCCCCTCTGAACCCCCAACACTCTGGCCCATCGGGGTGACGGATATCTGCTTTTTAAAAATTTTCTTTTTTTGGCCCATCGGGGCTTCGGATACCTGCTTTTTTTTTTTTTATTTTTCCTTGCCCATCGGGGCCTCGGATACCTGCTTTAATTTTTGTTTTTCTGGCCCATCGGGGCCGCGGATACCTGCTTTGATTTTTTTTTTTCATCGCCCATCGGTGCTTTTTATGGATGAAAAAATGTTGGTTTTGTGGGTTGTTGCACTCTCTGGAATATCTACACTTTTTTTTGCTGCTGATCATTTGGTGGTGTGTGAGTGTACCTACCGCTTTGGCAGAGAATGACTCTGCAGTTAAGCTAAGGGCGTGTTCAGATTGTGGAGGAAAAGTGGCCGCCATTTTAGACTTGCCGCATAACTCGGCTTAGGGCTAGTCGTTTGTGCTAAGTTAA

**Delta XB Deleted Sequence** (319 to 1120bp, Repeat A underlined)

CCGTGGATACCTGCCTTTTAATTCTTTTTTATTCGCCCATCGGGGCCGCGGATACCTGCTTTTTATTTTTTTTTCCTTAGCCCATCGGGGTATCGGATACCTGCTGATTCCCTTCCCCTCTGAACCCCCAACACTCTGGCCCATCGGGGTGACGGATATCTGCTTTTTAAAAATTTTCTTTTTTTGGCCCATCGGGGCTTCGGATACCTGCTTTTTTTTTTTTTATTTTTCCTTGCCCATCGGGGCCTCGGATACCTGCTTTAATTTTTGTTTTTCTGGCCCATCGGGGCCGCGGATACCTGCTTTGATTTTTTTTTTTCATCGCCCATCGGTGCTTTTTATGGATGAAAAAATGTTGGTTTTGTGGGTTGTTGCACTCTCTGGAATATCTACACTTTTTTTTGCTGCTGATCATTTGGTGGTGTGTGAGTGTACCTACCGCTTTGGCAGAGAATGACTCTGCAGTTAAGCTAAGGGCGTGTTCAGATTGTGGAGGAAAAGTGGCCGCCATTTTAGACTTGCCGCATAACTCGGCTTAGGGCTAGTCGTTTGTGCTAAGTTAAACTAGGGAGGCAAGATGGATGATAGCAGGTCAGGCAGAGGAAGTCATGTGCATTGCATGAGCTAAACCTATCTGAATGAATTGATTTGGGGCTTGTTAGGAGCTTTGCGTGATTGTTGTATCGGGAGGCAGTAAGAATCATCTTTTATCAGTACAAGGGACTAGTTAAAAATGGAAGGTTAGGAAAGACTAAGGTGCAGGGCTTAAAATGGCGATTTTGACATTGCGGCA
